# An Immune-Based Therapeutical Approach in an Elderly Patient With Fixed Cutaneous Sporotrichosis

**DOI:** 10.7759/cureus.53192

**Published:** 2024-01-29

**Authors:** Jesús Iván Martínez-Ortega, Samantha Franco-Gonzalez, Arely Gissell Ramirez Cibrian

**Affiliations:** 1 Dermatology, Dermatological Institute of Jalisco, “Dr. José Barba Rubio”, Jalisco, MEX; 2 Internal Medicine, XXI Century National Medical Center, Mexico City, MEX; 3 Medical Benefits, Mexican Institute of Social Security, Campeche, MEX

**Keywords:** thermally dimorphic fungi, fixed cutaneous, fungal infection, sporothrix schenckii species, cutaneous sporotrichosis

## Abstract

Sporotrichosis is a subcutaneous fungal infection caused by thermally dimorphic fungi from the *Sporothrix *genus, primarily prevalent in tropical regions of Latin America, Africa, and Asia. Mexico’s Jalisco state is an endemic hotspot with a remarkable prevalence rate of 54.4%. Clinical presentation varies based on immune status and virulence. The most common form is cutaneous-lymphangitic (67%), with fixed cutaneous cases accounting for 28%. This case study explores a traditional therapeutic approach for fixed cutaneous sporotrichosis but introduces a distinct immunological perspective.

## Introduction

Sporotrichosis is a subcutaneous fungal infection caused by thermally dimorphic fungi belonging to the *Sporothrix* genus. While the infection has a global distribution, the majority of cases arise from tropical and subtropical regions across Latin America, Africa, and Asia [1]. Within Latin America, countries such as Brazil, Colombia, El Salvador, Mexico, Uruguay, and Venezuela exhibit the highest estimated prevalence rates of sporotrichosis [2]. In Mexico, the state of Jalisco stands out as a prominent endemic region, accounting for a prevalence rate surpassing the national average. This region is hyperendemic with a prevalence rate reaching 54.4% in Jalisco, at a prevalence of 25 cases per 1,000 inhabitants [1-3]. The clinical manifestation of sporotrichosis varies depending on factors such as the patient’s immune status, the virulence of the causative agent, and the size of the initial inoculum. The most prevalent clinical presentation is cutaneous-lymphangitic (67%), while fixed cutaneous cases account for 28% of the total. In Mexico, potassium iodide is commonly used to treat most cases [2]. In this context, we present a case of fixed cutaneous sporotrichosis that was treated with a combination therapy approach. Furthermore, we delve into the molecular underpinnings that support the rationale behind this combined treatment strategy.

## Case presentation

**Figure 1:**
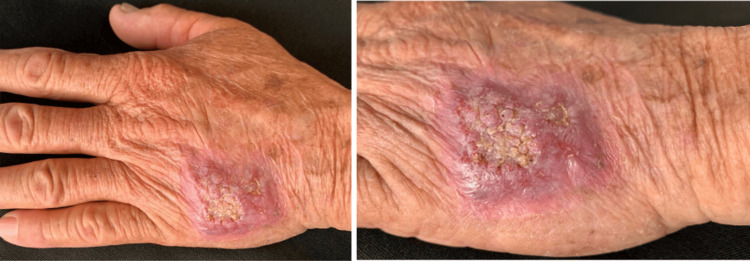
Clinical lesion on the dorsum of the hand. Isolated erythematous-violaceous plaque with a verrucous, yellowish center.

A 71-year-old male farmer from Cuquío, Jalisco, a known diabetic and hypertensive, developed a lesion on his left hand three months after a trauma in the field, which discharged purulent material but was not painful. Physical examination revealed isolated nodules on the dorsum of his hand, forming an erythematous-violaceous plaque with a verrucous, yellowish, keratotic center and poorly defined borders, measuring 3 x 3 x 0.5 cm (Figure 1).

**Figure 2:**
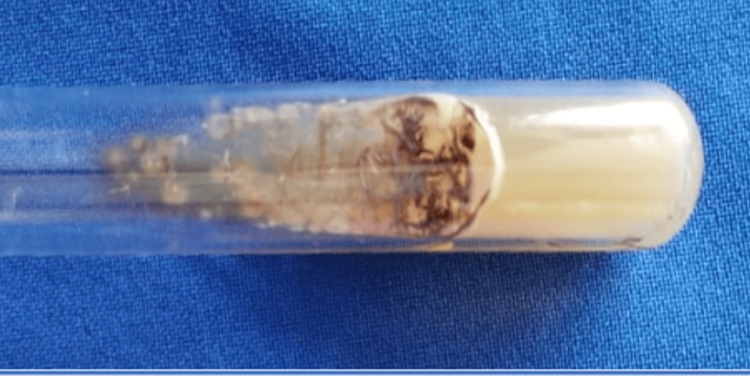
Mycological culture. Mycological culture shows colonies with a membranous appearance, beige color in the center, and brown pigment at the periphery.

Mycological culture displayed colonies compatible with *Sporothrix schenckii* (by phenotypic identification). On histopathological study, hematoxylin and eosin staining revealed evidence of pseudoepitheliomatous hyperplasia, intraepidermal neutrophilic abscesses, and a diffuse, dense inflammatory infiltrate in the dermis (Figure 2).

**Figure 3:**
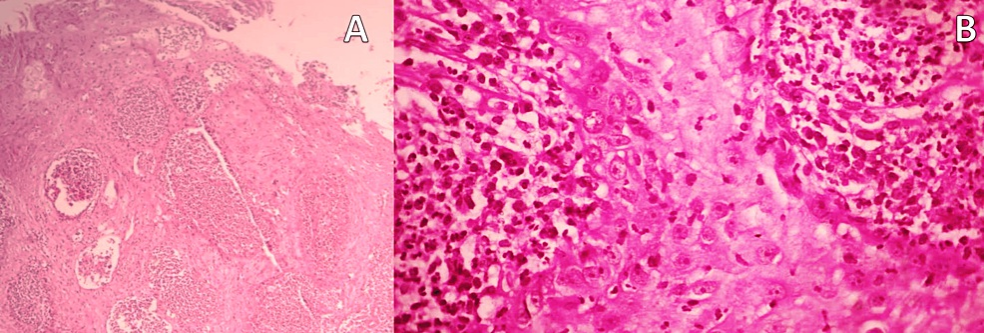
Histopathology. (A) Hematoxylin and eosin stain at 10× magnification showing pseudoepitheliomatous hyperplasia, intraepidermal neutrophilic abscesses, and a diffuse, dense inflammatory infiltrate in the dermis. (B) Magnified view at 40× of the inflammatory infiltrate within the dermis, depicting epithelioid histiocytes, plasma cells, neutrophils, and lymphocytes. This level of magnification enables a detailed observation of cellular and tissue characteristics in this sample.

A closer examination of the inflammatory infiltrate within the dermis revealed epithelioid histiocytes, plasma cells, neutrophils, and lymphocytes (Figure 3).

The diagnosis of fixed cutaneous sporotrichosis was established, and treatment was initiated with oral itraconazole at a dose of 200 mg/day. After three weeks, a complete resolution of the lesion was observed.

## Discussion

This case highlights an elderly farmer with diabetes, an immunosuppressive condition, residing in the most endemic area in Mexico for sporotrichosis. Considering the epidemiological history is essential for the suspicion of this condition and ruling out differential diagnoses. Mycological culture is necessary for the diagnosis [2-4].

The fixed or lymphocutaneous manifestation of sporotrichosis is influenced by factors pertaining to both the host and the microorganism. For instance, studies have indicated that the fixed presentation tends to exhibit fewer neutrophils compared to the lymphocutaneous form, and, in general, more immune cell infiltration and more inflammatory reaction [5,6]. This discrepancy is attributed to the ability of neutrophil-derived enzymes to break down the extracellular matrix, potentially facilitating the spread of the fungus through lymphatic vessels [5-7]. Overall, a more balanced immune response in fixed forms leads to better resolution and minimum tissue damage [6]. Additionally, strains of the fungus that can thrive at 35°C but not at 37°C, termed thermotolerant, are unlikely to disseminate through the host’s lymphatic system [8].

Neutrophils can eliminate the fungus in a concentration-dependent manner, but this efficacy is achieved when neutrophil levels are relatively elevated [9]. When pathogens invade the body, neutrophils employ three main strategies to eliminate them, namely, phagocytosis, degranulation, and the discharge of neutrophil extracellular traps (NETs) [10,11]. Research has demonstrated that the C-type lectin-like receptor on neutrophils, responsible for recognizing fungal antigens, can trigger the release of NETs [10]. This NET release is influenced by the size of microorganisms sensed by neutrophils [12]. Interestingly, a higher abundance of NETs has been observed in the lymphocutaneous form compared to the fixed form, potentially linked to the greater presence of neutrophils in the former [7]. Given the considerable size of the mycelial phase of *Sporothrix schenckii*, it may necessitate a regulated response from both neutrophils and NETs. Consequently, a larger initial inoculum of the pathogen would activate a higher number of neutrophils and induce NETs. As a consequence, an overly robust immune reaction might contribute to the dissemination of lymphocutaneous forms. Conversely, in fixed forms, a lower initial inoculum or a more balanced immune response would likely prevent such lymphocutaneous dissemination.

Gomes et al. compared the treatment durations of the two main clinical forms in their study, namely, lymphocutaneous and fixed cutaneous presentations. With investigations sharing similar epidemiological characteristics but focussing on a younger demographic, they found that patients exhibited a prolonged recovery period of approximately one month [13,14]. This observation supports the clinical implications of immunosenescence in the context of sporotrichosis infections.

Intriguingly, cryosurgery is known to possess immunostimulatory effects [14]. Further, the lesser-known ability of itraconazole to inhibit the sonic hedgehog pathway [15] and local immunosuppression [16] may contribute to immune enhancement, as supported by fungal models showing increased neutrophil activation against fungal infections, reduced Th2, and heightened Th1/Th17 cytokines [17]. Furthermore, the application of cryosurgery results in the destruction of the fungal structure, exposing otherwise non-exposed fungal cell molecules. One of these molecules, β-glucan, has been proven to exhibit immunoenhancing and immunomodulatory effects. Taken together, whether used in combination or alone, itraconazole and/or cryosurgery emerge as the most suitable options for older patients with fixed or lymphocutaneous forms of sporotrichosis [18,19].

**Figure 4:**
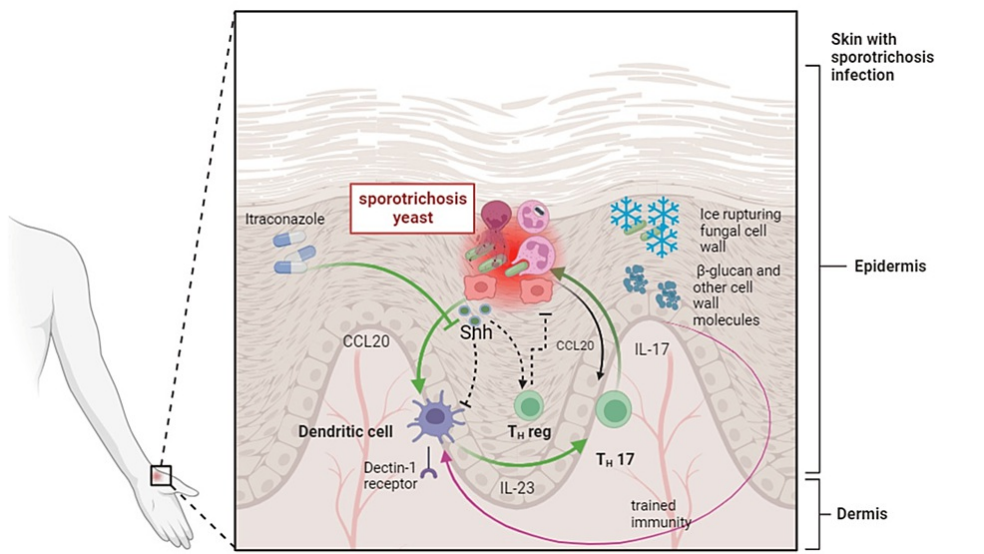
Immune responses and therapeutic immunomodulation in sporotrichosis. When keratinocytes are exposed to sporotrichosis, they generate cytokines that, through the IL23/17 axis, activate neutrophils. This activation leads to the elimination of the fungal infection through various mechanisms, including NETosis, phagocytosis, and enzymes. Simultaneously, keratinocytes produce Shh ligands that restore homeostasis, limiting inflammation by inhibiting the IL23/17 axis and activating regulatory lymphocytes. Administering itraconazole, an Shh pathway inhibitor, enhances immune stimulation by inhibiting this axis further. Conversely, during cryosurgery, ice crystals rupture the fungal cell wall and plasma membrane, exposing cell and plasma membrane molecules like β-glucan. This molecule exhibits immunostimulatory effects, contributing to immune activation. Image created using BioRender.

Other treatments appear to function through other mechanisms. For instance, potassium iodide, despite not exhibiting antifungal effects in vitro, is believed to exert antifungal activity in vivo through its conversion into iodine by neutrophil myeloperoxidase [20]. However, it is important to note that potassium iodide usage might pose concerns for certain elderly individuals who are already using angiotensin-converting enzyme inhibitors, potassium-sparing diuretics, or those with impaired kidney function, potentially leading to potassium toxicity [21]. Moreover, adverse effects such as gastrointestinal discomfort and thyroid disruptions may also arise [4]. Furthermore, hyperthermia has been documented to eliminate the fungi regardless of neutrophil/NET involvement, and both modalities may work as a non-immune-based therapeutic approach [9] (Figure 4).

## Conclusions

While adhering to conventional treatment for fixed cutaneous sporotrichosis in an older patient, our treatment approach considered immunological factors. We propose that the well-established antifungal effects of cryosurgery and itraconazole may not be the sole determinants of successful outcomes. Recognizing the importance of immunomodulatory effects becomes crucial when choosing sporotrichosis treatment, especially for patients with immunosenescence or other contexts of immunosuppression.
